# Factors contributing to healthcare professionals’ adaptive capacity with hospital standardization: a scoping review

**DOI:** 10.1186/s12913-023-09698-9

**Published:** 2023-07-26

**Authors:** Foteini Tsandila-Kalakou, Siri Wiig, Karina Aase

**Affiliations:** Centre for Resilience in Healthcare SHARE, Faculty of Health Sciences, University of Stavanger, N-4036 Stavanger, Norway

**Keywords:** Adaptive capacity, Healthcare professionals, Hospital standardization

## Abstract

**Background:**

Certain factors contribute to healthcare professionals’ adaptive capacities towards risks, challenges, and changes such as attitudes, stress, motivation, cognitive capacity, group norms, and teamwork. However, there is limited evidence as to factors that contribute to healthcare professionals’ adaptive capacity towards hospital standardization. This scoping review aimed to identify and map the factors contributing to healthcare professionals’ adaptive capacity with hospital standardization.

**Methods:**

Scoping review methodology was used. We searched six academic databases to September 2021 for peer-reviewed articles in English. We also reviewed grey literature sources and the reference lists of included studies. Quantitative and qualitative studies were included if they focused on factors influencing how healthcare professionals adapted towards hospital standardization such as guidelines, procedures, and strategies linked to clinical practice. Two researchers conducted a three-stage screening process and extracted data on study characteristics, hospital standardization practices and factors contributing to healthcare professionals’ adaptive capacity. Study quality was not assessed.

**Results:**

A total of 57 studies were included. Factors contributing to healthcare professionals’ adaptive capacity were identified in numerous standardization practices ranging from hand hygiene and personal protective equipment to clinical guidelines or protocols on for example asthma, pneumonia, antimicrobial prophylaxis, or cancer. The factors were grouped in eight categories: (1) psychological and emotional, (2) cognitive, (3) motivational, (4) knowledge and experience, (5) professional role, (6) risk management, (7) patient and family, and (8) work relationships. This combination of individual and group/social factors decided whether healthcare professionals complied with or adapted hospital standardization efforts. Contextual factors were identified related to guideline system, cultural norms, leadership support, physical environment, time, and workload.

**Conclusion:**

The literature on healthcare professionals’ adaptive capacity towards hospital standardization is varied and reflect different reasons for compliance or non-compliance to rules, guidelines, and protocols. The knowledge of individual and group/social factors and the role of contextual factors should be used by hospitals to improve standardization practices through educational efforts, individualised training and motivational support. The influence of patient and family factors on healthcare professionals’ adaptive capacity should be investigated.

**Trial registration:**

Open Science Framework (https://osf.io/ev7az) https://doi.org/10.17605/OSF.IO/EV7AZ.

**Supplementary Information:**

The online version contains supplementary material available at 10.1186/s12913-023-09698-9.

## Background

Studies have shown discrepancies between hospital policies and procedures set to improve quality of care and their implementation by healthcare professionals [1, 2]. Why do healthcare professionals not comply with institutional policies, protocols, guidelines, and checklists set to improve quality? Healthcare professionals may be well intentioned and strive to offer quality of care, but they also face challenges such as limited resources, increasing work pressure, and burnout [3–5]. Non-compliance is multifactorial due to the complexity of the healthcare system and the quantity of information and hospital policies. Some factors are linked to the individual healthcare professionals, e.g. training, beliefs, habits, psychological factors and other factors are contextual such as social norms, staff workload and competing goals between the individual and the institution.

Adaptation or adaptive capacity is seen as a main pillar in resilience across several disciplines [6–8]. Several studies have aimed at exploring and understanding how resilience contributes to healthcare professionals’ adaptive capacities towards challenging work conditions [9–11], but still it is poorly understood. According to Smaggus [2] hospital healthcare professionals proactively adapt to compensate for systemic problems such as protocols and technology poorly aligned with their tasks. They do so through their dedication, expertise, and creativity. However, these adaptations might come at a cost to professionals’ well-being as they often include working longer and more intense hours. In the context of this study healthcare professionals’ adaptive capacity is seen as essential for hospital standardization to be successfully practiced thus contributing to quality of care. Adaptations might come in the forms of compliance or non-compliance to standardized guidelines and protocols, or in the forms of adjustment of the contents of the standardization efforts.

There are certain factors that contribute to healthcare professionals’ adaptive capacity such as habits, stress, anxiety, burnout, coping mechanisms, motivation (internal and external), intention, level of knowledge and education, cognitive capacity, perceptions, attitudes, and beliefs (individual and social) [9–11]**.** No evidence was found of literature reviews exploring factors that contribute to healthcare professionals’ adaptive capacity with hospital standardization, except one focusing on hand hygiene guideline adherence [12] and one on nurses’ non-compliance in infection prevention [5].

Therefore, the aim of this scoping review is to identify and map the factors contributing to healthcare professionals’ adaptive capacity with hospital standardization. Specific research questions addressed by this review were:

- 1. In which hospital standardization practices have healthcare professionals’ adaptive capacity been studied?
- 2. What factors influence healthcare professionals’ adaptive capacity with hospital standardization and how can they be categorized?

## Methods

A scoping review methodology was chosen because it provides a transparent approach to mapping relevant literature in emerging fields or topics [13, 14] and has a broader “scope” and more expansive inclusion criteria than a systematic review [15, 16]. It also allows for studies using different designs and methods to be included and synthesized, which was considered necessary for this review. We followed the methodological stages outlined by Arksey and O’Malley [13] and Levac et al.’s [17] enhancement to conduct the review. These were: (1) Identifying the research question, (2) Identifying relevant studies, (3) Study selection, (4) Charting the data, (5) Collating, summarizing and reporting the results, and (6) Consulting with relevant stakeholders. A review protocol was developed according to Peters et al. [14] and registered on October 11^th^ 2021 on the Open Science Framework (https://osf.io/ev7az) https://doi.org/10.17605/OSF.IO/EV7AZ. The reporting of the review follows the PRISMA-ScR Checklist [18] (Additional file 1).

### Eligibility criteria

Articles were assessed against the following inclusion criteria: English-language, peer-reviewed research articles of any type published in scholarly journals where the full text was available, as well as grey literature not published in peer-reviewed journals. We chose to focus on healthcare professionals above 25 years indicating that they would have a minimum level of clinical experience including experiences with hospital standardization efforts. Furthermore, we chose the hospital setting to be able to possibly compare different standardization efforts identified. For a full description of inclusion and exclusion criteria, see Additional file 2.

### Information sources

The focus of the review was on peer-reviewed literature and electronic databases from different disciplines such as biomedicine, psychology, health services research, and nursing were searched on 12.10.2021 to identify relevant studies. The electronic databases searched included Scopus, MEDLINE (Ebsco & Ovid), Web of Science, CINAHL, EMBASE and PsycINFO [19]. Search terms were discussed by the three authors. The preliminary search strategy, search terms and inclusion/exclusion criteria were checked by a research librarian at the University of Stavanger.

The electronic database searches were then conducted by one researcher (FTK) and included all citations published before October 2021. A combination of Title, Subject, Subject headings, MeSH terms, and Keywords/Text words was used. The search strategy was adapted to individual databases. An example of a search strategy is presented in Table 1.

**Table 1:** Search strategy used in EMBASE

**Multifield search**
**Limits:** Human, English language, publication type** article**
**Title search**
(adherence OR compliance OR noncompliance OR comply) **AND** (improvement strategy OR guideline OR protocol OR regulation OR standard OR rule OR law OR policy OR procedure OR recommendation OR routine) **AND** (doctor OR physician OR nurse OR staff OR clinician OR healthcare professional OR provider OR practitioner)
**Subject Headings search**
(protocol compliance) **AND** (practice guideline OR clinical practice OR health care practice OR clinical protocol) **AND** (physician OR nurse OR nurse practitioner OR health care personnel)
**Keyword Heading & Text word search**
(resilien* OR coping OR adapt*)

To reduce the likelihood that relevant articles were overlooked we also hand searched reference lists of included articles and did an additional snowball-search. To further ensure that all relevant information was captured we conducted a targeted search of the grey literature in Google Scholar and in the following grey literature databases: Grey Literature Report (https://www.greylit.org/) and OpenGrey (http://www.opengrey.eu/). Hand searches, snowball-search, and grey literature resulted in an additional 19 records. All the search results were imported into EndNote bibliographic software and merged.

### Selection of sources of evidence

The review process consisted of three levels of screening: (1) title, (2) abstract, and (3) full text. For the first level of screening, one researcher (FTK) screened the titles of retrieved citations. Abstract and full text screening involved two researchers (FTK and KA) who shared and independently assessed the articles to determine if they met the inclusion/exclusion criteria. Articles considered relevant by the reviewers were included in the full-text review. Discrepancies about study eligibility at the full-text review stage was solved through discussion for 14 studies. Consensus was achieved between the two researchers making it unnecessary to involve the third researcher (SW) at this stage.

Quality assessment of the included studies did not form part of the current scoping review [13]. Therefore, all studies were included in the analysis as they would potentially contribute to mapping the knowledge base.

### Data charting process and data items

An electronic data charting form was developed in excel to guide data charting from included articles. Data concerning study characteristics, e.g., authors, year of publication, and the methodology, e.g., design, data collection, participants, results were charted in addition to information related to the aim of the review, i.e., hospital standardization type, factors of adaptive capacity, individual level, group/social level, and reviewers’ notes (Table 2).

**Table 2:** Overview of charted data items

Charted Data Items	
a.	Author (s)
b.	Publication Year
c.	Country of origin
d.	Adherence to standardization practice
e.	Study type
f.	Study design
g.	Study purpose
h.	Setting
i.	Sample size
j.	Participants
k.	Results
l.	Individual factors
m.	Group/social factors
n.	Reviewers’ notes
o.	Full reference

The initial data charting sheet was validated by two reviewers (FTK and KA) with three articles each to corroborate consistency, as recommended by Daudt, van Mossel and Scott [20]. All data was extracted by two researchers (FTK, KA) independently and then agreed and merged with input and discussion by the third researcher (SW).

### Synthesis of results

Results were synthesised and presented using frequency counting as well as summarised in text as categories. The data were compared and synthesised to summarise study characteristics, hospital standardization effort, and factors affecting healthcare professionals’ adaptive capacity. All three authors were involved in the process of synthesizing and describing the results in a suitable format.

### Consultation with stakeholders

According to Levac et al. [17], we conducted the optional stage of consulting with relevant stakeholders once the results were synthesized. The researchers of the Centre for Resilience in Healthcare SHARE, University of Stavanger were identified as relevant and knowledgeable stakeholders on the topic. Hence, an email with draft results was sent to all 79 centre researchers with a request for feedback and input on any additional sources of information relevant to the research questions of the scoping study. Stakeholders’ literature input was sent to author FTK (one book and three studies) who then assessed the information. No additional studies were included.

## Results

The search resulted in 1414 unique records of which 180 full-text articles were assessed for eligibility and 57 were included in the review [12, 21–76]. From the grey literature four articles met the inclusion criteria. The most common reasons for exclusion were no healthcare professional related factors described, mixed sample with unclear reporting of results for healthcare professionals, not in hospital setting or setting unclear, or not a healthcare professional sample.

**Fig. 1:**
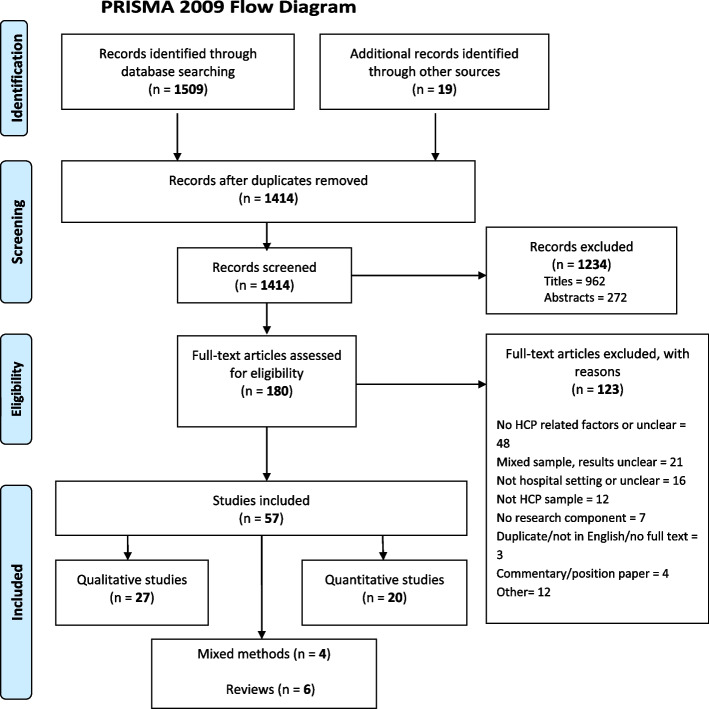
PRISMA flow diagram

A total of 27 qualitative studies, 20 quantitative studies, six literature reviews, and four mixed-method studies were included in the review. Figure 1 demonstrates the inclusion and exclusion of records at each stage of the screening process, using the PRISMA flow diagram [77].

### Characteristics of studies

The included studies were published between 2000 and 2021, with a tendency towards increased publication frequency over the last five-year period (2017–2021). The studies originated from 26 different countries with all continents represented. Several of the studies were conducted in Australia (*n* = 5), Jordan (*n* = 5), USA (*n* = 5), the Netherlands (*n* = 4), China (*n* = 3), and the UK (*n* = 3). Twenty-nine studies were conducted in developed countries while 19 in developing countries. A total of 9 studies had unspecified country origin, the reason usually being that they were literature reviews.

### Hospital standardization practices

The review identified healthcare professionals’ adaptive capacity in numerous standardization practices across hospitals’ specialties. The most common standardization types were clinical or practice guidelines and protocols, precautions, procedures, checklists, policies, forms, and hospital-generic precautions, rules, and regulations. The most common areas of standardization were hand hygiene and infection prevention, personal protective equipment use, surgery, medication administration, cancer, mother and new-born care, falls, asthma, and pneumonia (Table 3). Three studies on personal protective equipment use were conducted during pandemics [51, 52, 54] while two studies explored infection prevention after pandemics [32, 55].

**Table 3:** Hospital standardization practices

Standardization practice area	Studies (n = 57)
Infection prevention and control	*n* = **14** [12, 21–33]
Hospital-generic (incl. national, internat.)	*n* = **10** [34–43]
Single disease-specific (e.g., asthma)	*n* = **7** [44–50]
Personal protective equipment use	*n* = **6** [51–56]
Surgery	*n* = **5** [57–61]
Cancer	*n* = **3** [62–64]
Mother & new-born care	*n* = **3** [65–67]
Medication administration	*n* = **2** [68, 69]
Falls	*n* = **2** [70, 71]
Others	*n* = **5** [72–76]

### Factors influencing healthcare professionals’ adaptive capacity

The factors influencing healthcare professionals’ adaptive capacity with hospital standardization practices were grouped in eight categories as described in Table 4. The eight categories constitute a combination of individual and group/social factors deciding whether hospital healthcare professionals comply with or adapt hospital standardization practices. Below the eight categories are described in more detail.

**Table 4:** Factors contributing to healthcare professionals’ adaptive capacity

Factors	Description
*1. Psychological and emotional*	Healthcare professionals’ confidence, invulnerability, feelings, and justification towards hospital standardization. E.g., high levels of confidence with treatment choices would lead oncologists to adapt the recommendations for cancer pain management [63]
*2. Cognitive*	Healthcare professionals’ attitudes and beliefs towards hospital standardization. E.g., nurses justifying adaptations when they believe that rules and policies are not in the best interest of the patient [75]
*3. Motivational*	Healthcare professionals’ moral responsibility, obligation, and personal comfort with hospital standardization. E.g., physicians stating that following tuberculosis infection control practices is a moral responsibility [33]
*4. Knowledge and experience*	Healthcare professionals’ individual knowledge, education, and experience with hospital standardization. E.g., nurses increased knowledge and clinical experience with standard precautions showed a positive correlation with adaptations [42, 29]
*5. Professional role*	Healthcare professionals’ authority, autonomy, and clinical judgement related to hospital standardization. E.g., nurses using discretionary judgement to selectively make decisions about when to enforce a strict interpretation of the hospital policy for surgical count or not [59]
*6. Risk management*	Healthcare professionals’ self-protection and safety precautions related to hospital standardization. E.g., wanting to protect themselves from the personal costs of getting infections [41] or being reprimanded or reported [71]
*7. Patient and family*	Healthcare professionals’ desire to meet patient and family needs and expectations, and to protect them. E.g., reduced use of facemasks to prevent patients feeling stigmatized or isolated [24]
*8. Work relationships*	Healthcare professionals’ collaborative environment, collegial support, communication, and teamwork. E.g., teamwork and collegial support influencing how successful the use of fall prevention guidelines are [71]

#### Psychological and emotional factors

In many instances professionals choose to adhere to infection prevention guidelines due to the psychological pressure or fear of contracting or spreading infections [23, 32, 41]. Adverse incidents with infection prevention equipment are perceived as stressful and worrying [51], while following standard precautions would decrease their anxiety [41].

Healthcare professionals’ sense of invulnerability and confidence would lead them to adapt hospital standardization [22, 31, 55, 63, 75]. Reasons would differ from physicians considering themselves to be entitled to work independently without protocols to guide them [31], to professionals feeling minor concerns for infection transmission over time when not acquiring any infection [55] to nurses expressing psychological gratification about their own ability to creatively solve problems and work around standardization practices  [75].

#### Cognitive factors

Various attitudes and beliefs were reported in several studies as influencing healthcare professionals’ adaptive capacity or compliance to hospital standardization [28, 29, 33, 37, 41, 45, 47, 48, 52, 55, 61, 62, 69, 73, 75]. Some of these attitudes and beliefs were patient related [41, 45, 52, 62, 69, 74, 75]. For example, in a study on the use of personal protective equipment in the emergency room during the COVID-19 pandemic, there was only a slight difference between healthcare professional beliefs on whether the equipment was protective or not for patients (52% vs. 46%) [52]. In another study, physicians believed that diarrhoea was a low-risk disease with overrepresentation among poor people but adapted the diarrhoea treatment to patients with higher social status [45]. Moreover, nurses justified adaptations when they believed that use of gloves or masks was an exaggeration when treating children considered low risk and not contagious [41].

Attitudes and beliefs were also related to the professional group that healthcare professionals belonged to [29, 61]. Physicians and nurses had opposing views on surgical count protocol violations and what constitutes safe clinical practice [61]. Nurses believed that physicians had lower hand hygiene compliance, while physicians believed that they were role models and leaders of hand hygiene and would warn other staff members [29].

Healthcare professionals’ agreement or disagreement with specific guidelines also led to adaptations of hospital standardization. Adaptations were made when for example radiologists considered diagnostic imaging guidelines not useful, too rigid, or that they failed to include specific information about changes [47] or were perceived as inefficient or unnecessary [75], not relevant for the clinical practice [37, 64], or not relevant for certain care systems [48], or when healthcare professionals doubted the effectiveness of isolation precautions to prevent disease contagion [55]. Similarly, a systematic review reported adaptations when guidelines and other standardized practices were considered too generic, promoted 'cookbook medicine', oversimplified, difficult or controversial treatment decisions, or when the evidence they were based on was conflicting [62]. By contrast healthcare professionals complied to standards, protocols and guidelines when believed to be useful tools [49] in clinical decision making and providing uniform care [48, 62], were easy to understand, highly relevant to clinical practice and patient population, and based on credible information sources [62].

#### Motivational factors

Motivational factors for healthcare professionals’ adaptive capacity are mainly reported in studies on infection prevention and control. Personal motivational drivers such as moral responsibility, obligation and duty are reported by different professional groups related to infection control practices in the emergency room and on hospital wards in general [26, 33], within tuberculosis infection control measures [22], within hand hygiene obligations [29], within venous thromboembolism prophylaxis management [46], within respiratory infectious diseases [24], and with clinical practice guidelines to prevent falls and injuries [71]. The moral responsibility would be directed towards themselves as professionals to reduce transmission of pathogens or expressed as a duty of care to their patients. Motivational factors were most often internally driven in the sense of professionals’ own intent and feelings of psychological safety [30, 75], while some studies reported on external drivers such as a motivational person in their organization [71], the intensity of activity in the clinical setting [30] or organizational neglect of occupational health and safety [60].

Healthcare professionals’ comfort or discomfort with personal protective equipment would influence their motivation to adapt infection prevention and control standardization practices [24, 33, 41, 51, 53, 56].

#### Knowledge and experience factors

Knowledge and training of standardization practices was described as important and increased compliance among nurses [34, 42, 43] and younger physicians [55], but was not seen as sufficient for physicians and nurses in other studies [25, 44, 47, 50, 55, 58, 59].

Length and type of clinical experience would often lead healthcare professionals to either adapt hospital standardization [35, 41, 55, 59, 61, 67, 73] or to comply with it [43, 68, 71]. For example, experienced senior nurses had more confidence to adapt protocols in intensive care units [59] or during fever management [73], than less experienced nurses with barcode medication administration technology [68]. Among surgical team members, physicians relied on their experience and tactical knowledge [61], nurses on their repeated experience of working daily with the same instrument trays [59] and disregarded surgical count guidelines or made workarounds on surgical safety checklist use [58]. However, in another study increased length of experience was reported as a contributing factor to compliance with hand hygiene for both physicians and nurses [43].

Insufficient knowledge and training led healthcare professionals to make adaptations. For example, knowledge deficits about tuberculosis led healthcare professionals to use ineffective measures in preventing transmission [33]. Similarly, midwives’ limited knowledge of aspects of infection prevention control guidelines [23] or being unaware that national postnatal care protocols had been updated led them to make adaptations based on inappropriate experiential knowledge [60].

For some healthcare professionals, negative experiences during clinical practice increased their compliance with clinical guidelines [71], while experience of lack of consequences led them to continue their adaptations of universal precautions [55].

#### Professional role factors

In many cases, the clinical role or profession of healthcare workers influenced their ability and desire to adapt hospital standardization [12, 55, 57, 60, 75]. Nurses defined problem solving as part of their job thus contributing to workarounds from standardization practices [75]. Physicians often defined their role in authoritative ways contributing to lower compliance with hospital standardization than other professions, for example within hand hygiene [12], the surgical safety checklist [57, 60], and MRSA precautions [55]. In one study, professional status and reputation were identified to influence physicians’ clinical decision-making [45]. Healthcare professionals’ perception of their own roles also challenged their possibility to intervene in each other’s work tasks and their ability to collectively adapt standardization practices [45, 57].

Furthermore, autonomy and clinical and/or professional judgment were seen as vital elements of healthcare professionals’ adaptive capacity [39, 59, 62, 75]. For example, commitment to infection prevention and control was high in a neonatal unit, however, severely constrained resources made improvisation a vital element of professionals’ clinical judgment and adaptive capacity [23].

#### Risk management factors

Healthcare professionals adapted their practices to meet hospital standardization due to individual perceptions of risk and belonging personal costs. They adhered to infection prevention guidelines to protect themselves from being infected or from infecting family and others [24, 27, 29, 33, 41, 53, 56], or they wanted to avoid reprimands and litigations [46, 62, 72] or negative media attention [71]. The perceived risk for reprimands or litigations might for example lead to nurses performing fall prevention according to the guideline “just in case” even with non-risk patients [71] p90. The same goes for physicians sending patients for x-rays “just to be safe” [71] p90. However, in another study perceived enforcement of rules in the form of monitoring and threats of punishment or sanctions had no direct or indirect effect on physicians’ compliance [40].

Clinical practice guidelines were adapted or disregarded if healthcare professionals perceived them to constitute a potential risk to patients [59, 67, 69, 75]. This could involve physicians using more highly concentrated medications than recommended to prevent fatal arrhythmia [69] or nurses to disregard the protocol for surgical count of instruments in life-threatening emergencies [59].

#### Patient and family factors

The main reason for healthcare professionals wanting to adapt hospital standardization was to meet patient needs. In general, this involved deviations from hospital guidelines or policies when they saw them as barriers to patient care and/or patient safety. Patient needs were exemplified as timely care, patient-centred care, quality of patient communication, privacy, improved outcomes [36, 55, 56, 67, 74, 75] and customized care [62–69]. Several studies related to infection prevention including three during pandemic situations pointed at adaptations made to personal protective equipment protocols to improve patient communication, reduce patients’ feeling of isolation, and better establish therapeutic relationships [22, 52, 54–56]. This was especially relevant for older patients [55] and children [41, 56]. In emergencies, workarounds of protocols were justified not to jeopardise the patients’ safety [75], while in surgical settings compliance with the checklist protocol was seen as preserving patient safety [60].

Family factors were related to presence and expectations, and cultural conflicts. Examples of adaptations span from clinicians not complying with the family witnessed resuscitation protocol as they value it as traumatic for relatives with risk of PTSD [72] to pressure for antibiotics and intravenous fluids in diarrheal management [45] to disapproval of pre-operative skin preparation policy due to cultural preferences [76].

#### Work relationship factors

Most studies reporting on work relationship factors were related to conditions negatively affecting the adaptive capacity of healthcare professionals such as power issues, group norms, hierarchical relationships, and breakdown in communication [21, 23, 31, 41, 57, 59, 61, 65]. This could entail surgeons’ power influencing the practice of the surgical count procedure negatively where nurses felt unable to demand to undertake the count even though it constitutes a crucial safeguard for the outcome of the surgery [59, 61]. Hierarchical relationships were shown to negatively affect the use of the safe surgery checklist as surgeons and anaesthetists would disincline to volunteer information and openly communicate with each other and other team members [57]. Breakdown in communication was identified to negatively influence healthcare professionals’ adaptive capacity within antimicrobial prophylaxis [21], postnatal care protocols [60], and infection outbreaks [55].

A few studies reported on positive effects of work relationship factors such as peer pressure in the forms of healthcare professionals’ reminding each other to wear protective equipment [24], physicians acting as positive role models to other staff members on hand hygiene [29], nurse leaders modelling practicing safety rounds to staff [38] or collegial support from senior medical and nursing staff to junior professionals to improve adherence to standardization practices in the emergency department [70].

### Contextual factors influencing healthcare professionals’ adaptive capacity

Based on our synthesis of studies we identified several contextual factors that influenced healthcare professionals’ adaptive capacity with hospital standardization. These were factors “outside” the individual and group/social level. Even though the review did not focus specifically on the organizational or institutional level, the contextual factors formed parts of healthcare professionals’ explanations for degree of adaptation or compliance with hospital standardization.

#### Guideline “system”

Some studies described characteristics of the guidelines per se to influence how healthcare professionals adapted to them or not [24, 47, 48, 68, 71]. For example, guidelines that were too long and ambiguous or outdated and unclear [24, 47] or complex [64] were considered as barriers as healthcare professionals were confused and unsure how to adhere to them. Moreover, constantly changing guidelines given the time restrictions of daily clinical practice overwhelmed healthcare professionals who could not keep up with the updates or changes [24]. In addition, insufficient guidelines which lacked specific information were seen as a barrier and practical implementation depended on the healthcare professionals’ expertise [48].

However, high usability and guidelines that reflected national or international guidelines facilitated healthcare professionals’ compliance [24, 68].

#### Cultural norms

Workplace culture was described to influence adaptation or compliance with hospital standardization [24, 33, 41]. For example, adaptations were made when standard precautions were not the routine practice in the clinical department [41], when there was complacency to infection prevention control guidelines [24] or when workplace culture was part of a national culture [33]. When hospital standardization practices were followed by senior colleagues [41] or by all staff the compliance was high [24].

#### Leadership support

Several studies reported that the level of adaptation or compliance with hospital standardization was influenced by the level of support healthcare professionals received by their clinical leadership [24, 29, 33, 60, 62, 65, 71]. Leadership support was understood as visibility, encouragement, and modeling compliance with standardization practices [24, 29, 38, 65].

#### Physical environment

Healthcare professionals described various factors in the physical environment that led to adaptations of hospital standardization practices [24, 45, 62, 67, 71]. For example, limited access to treatment services and facilities [62], wards being too crowded, noisy, and dirty [45], lack of adequate ventilation, isolation rooms, and shower facilities to prevent infection transmission [24], narrow hospital bathrooms [71], or lack of vital space in examination cubicles [60].

#### Time

Time constraints were in several studies described as a reason for adaptations [29, 36, 48, 49, 57, 58]. For example, during emergencies there was no time to either perform proper hand hygiene or proper use of gloves [29], or to perform time-out procedures or safety checklists during surgical operations [57]. However, a systematic review suggested that implementation of the surgical safety checklist reduced time delays as miscommunication and confusion were avoided [60].

#### Workload issues

Increased workload was mainly reported in studies on infection prevention practices to explain healthcare professionals’ adaptations of hospital standardization [12, 21, 24, 29, 55]. Similarly, in a study on perinatal care obstetricians reported that they were more likely to comply with changes in practice if their workloads did not increase [34]. Staff shortages leading to demanding workloads was also a contributing factor for midwives to collectively decide not to update their knowledge of the new post-natal care protocols, despite training being offered [60].

## Discussion

In this paper we have reviewed the literature to identify the factors contributing to healthcare professionals’ adaptive capacity with hospital standardization. We have documented that adaptive capacity is multidimensional according to eight factors: psychological and emotional, cognitive, motivational, knowledge and experience, professional role, risk management, patient and family, and work relationships. This multidimensional aspect is supported by Huey and Palaganas’ [11] emphasizing the influence of individual and workplace cultural factors. Individual traits such as having a higher purpose is in our review specified as motivational, emotional, cognitive, and knowledge-based factors. Our review also adds group/social factors including work relationship, professional role, and physical environment, in line with Toode, Routasalo and Suominen [78]. New in this study is the establishment of the patient and family factor as a main driver for healthcare professionals’ adaptation of hospital standardization.

The eight factors of adaptive capacity are situated within a contextual setting, described by healthcare professionals as the background for their adaptation. Time and workload issues were most frequently described in studies on infection prevention and control and in surgery, with different reasoning. The time issue in infection prevention and control is related to the time-consuming and resource intensive procedures, while in surgery the time issue is related to urgency and acute situations. Both contexts might lead to a need for healthcare professionals having to adapt protocols and guidelines. Individual factors are indisputable engrained in the contextual surroundings meaning that healthcare professionals’ adaptive capacity needs to be understood in light of the guideline system, cultural norms, leadership support, time and workload issues, and the physical environment. This is in line with previous research on the role of context in healthcare [79, 80].

This scoping review covers 26 different countries representing all continents of which 29 studies are from developed countries and 19 from developing countries. We did notice some variation in the extent and type of details reported by healthcare professionals across regions and countries. However, a geographic comparison was not included in our scope and future research comparing continents, regions, or countries based on their economic status and healthcare professionals’ adaptive capacity should be conducted.

In our review infection prevention and control and practices related to hand hygiene and use of personal protective equipment stand out as the most common standardization practices studied. This is an area with clear individual and organizational targets thus requiring a combination of individual and organizational adaptive capacity [81]. Based on our review and previous research we claim that the field of adaptive capacity and resilience would benefit from incorporating knowledge on individual factors to succeed [82–84]. Adaptive capacity for healthcare professionals and healthcare organizations seems to depend on each other more than the research has acknowledged so far and should be the topic of future research.

### Implications for hospital management and practice

The new knowledge from this review on individual, group/social factors and contextual factors influencing healthcare professionals’ adaptive capacity should be used by hospitals to improve and tailor make current standardization practices. Efforts should be made to construct educational efforts, individualise training and motivational support, and to address the role of patients and families as the main driver for healthcare professionals’ adaptation of hospital standardization.

Based on the findings of this scoping review, complex standardisation practices should be revised to be easily understandable, as short as possible, and relevant to the professional practice. Healthcare professionals should be involved in standardization development and/or revisions to achieve this. Educational efforts to inform healthcare professionals on new or revised standardisation practices should integrate knowledge building not only on the standardisation measure in itself, but also on how individual, group/social, and contextual factors promote or hamper their compliance to or adaptation of it. This is especially important within the areas of infection control and personal protective equipment as the evidence for healthcare professionals’ adaptation is strong.

### Strengths and limitations

The review was conducted in accordance with an acknowledged framework for scoping reviews and the Preferred Reporting Items for Systematic reviews and Meta-Analyses extension for Scoping Reviews (PRISMA-ScR) guidelines. The scoping methodology allowed information from a broad range of studies, using different designs and methods, to be included and synthesized. The findings highlight individual and group/social factors for healthcare professionals to comply with or adapt hospital standardization practices.

The review was limited to English-speaking literature and the included studies were not assessed for quality. The review does not provide a definitive account of the successful outcomes of healthcare professionals’ adaptive capacity towards standardization practices. Moreover, this scoping review focused on individual factors and group/social factors and did not include a full review of organizational factors as this is done in other studies. Finally, it was not possible to draw any conclusions on how pandemics influence healthcare professionals’ adaptive capacity, as only five studies met the inclusion criteria. This should be followed-up with further research.

## Conclusions

Our study identified the following hospital standardization practices where healthcare professionals’ adaptive capacity has been studied: clinical guidelines and protocols, precautions, procedures, checklists, policies, forms, and hospital-generic precautions, rules, and regulations. These are typically studied within infection prevention and control, followed by more disease-specific areas such as for example cancer.

There has been lack of knowledge on factors impacting on healthcare professionals’ adaptation or compliance with hospital standardization. This scoping review stands out by identifying a multidimensional set of eight factors at the individual and group/social level. The main factor influencing healthcare professionals’ adaptation of hospital standardization was patient and family needs. The review also identified surrounding contextual factors influencing healthcare professionals’ adaptive capacity including the guideline system, cultural norms, leadership support, physical environment, time, and workload issues.

Future research needs to investigate the relationship between individual factors for adaptive capacity and their contextual setting, as well as the relationship between individual, group/social, and organizational factors.

## Supplementary Information

**Additional file 1.** Preferred Reporting Items for Systematic reviews and Meta-Analyses extension for Scoping Reviews (PRISMA-ScR) Checklist.

**Additional file 2.** Inclusion Exclusion criteria.

**Additional file 3.** Table of all studies included in the review.

## Data Availability

The datasets used and/or analyzed during the current study are available from the corresponding author on reasonable request.

## Abbreviations

PRISMA-ScR: Preferred Reporting Items for Systematic reviews and Meta-Analyses extension for Scoping Reviews
MeSH: Medical Subject Headings
MRSA: Methicillin-resistant *Staphylococcus aureus*
PTSD: Post traumatic stress disorder
