# Supplementary material for: Factors contributing to healthcare professionals’ adaptive capacity with hospital standardization: a scoping review
Source: BMC Health Serv Res. 2023 Jul 26;23:799. doi: 10.1186/s12913-023-09698-9 (PMC10369840; doi:10.1186/s12913-023-09698-9)
Supplement: Supplementary file 2 — Additional file 2. Inclusion Exclusion criteria. [file 12913_2023_9698_MOESM2_ESM.docx]

**Additional file 2**

| **Inclusion Criteria** | |
| --- | --- |
| Types of studies | - Any publication dates - Any geographical location - English Language - Any quantitative study (RCT, non RCT, observational, cohort, case control) - Any review (systematic, narrative, qualitative) - Any qualitative and mixed methods studies - Full text - Peer reviewed - Grey literature/not published in a peer reviewed journal |
| Types of participants | - Adults (>25 years) - Healthcare professional with experience |
| Setting/context | - Hospital, secondary/specialist care |
| Types of phenomena & interventions | - Any article with information about coping, adaptation, compliance, noncompliance, adherence - Any hospital standardization effort, guidelines, procedures, improvement strategies linked to clinical practice |
| Types of  components | - Any information about psychological factors, habits, stress, anxiety, coping, distress, motivation, intention, knowledge, education, cognition, attitudes, beliefs, perceptions, behavior |

| **Exclusion Criteria** | |
| --- | --- |
| Types of studies | - Not in English Language - Editorials, commentaries, case reports, letters (written to convey opinion or stimulate research /discussion, with no research component) - Conference and other proceedings, abstracts, protocols |
| Types of participants | - Children and youth, adults <25 yrs. - Pharmacists and pharmacy staff, not healthcare professionals, healthcare students and healthcare students in training |
| Setting/context | - Any healthcare outside the hospital setting provided in primary healthcare like e.g., hospice care, nursing home, community care, home or rehabilitation care, general practice etc. |
| Types of phenomena & interventions | - Any hospital practice not involving a standardization effort - Any hospital standardization effort for conducting research - Any hospital practice due to insurance policies |
