# Supplementary material for: Factors contributing to healthcare professionals’ adaptive capacity with hospital standardization: a scoping review
Source: BMC Health Serv Res. 2023 Jul 26;23:799. doi: 10.1186/s12913-023-09698-9 (PMC10369840; doi:10.1186/s12913-023-09698-9)
Supplement: Supplementary file 3 — Additional file 3. Table of all studies included in the review. [file 12913_2023_9698_MOESM3_ESM.docx]

| **Additional file 3- Table of Included studies** | | | | | |
| --- | --- | --- | --- | --- | --- |
| **Author, year, reference** | **Country** | **Standardization type** | **Study design** | **Individual factors** | **Group/Social Factors** |
| Erasmus et al 2010 [12] | N/A | Hand hygiene | Systematic review | professional group (doctor), risk, | performance feedback, workload |
| Bonfait et al 2010 [21] | France | Infection prevention and control | Survey | negligence, training | communication, workload |
| Chapman et al 2017 [22] | Domenican Republic | Infection prevention and control | Semi structured interviews & focus group interviews | moral duty, invincibility, confidence, distress/powerlessness,  patient needs, family-presence | context restrictions |
| Herbec et al 2020 [23] | Zimbabwe | Infection prevention and control | Case study with interviews and ethnographic observations | awareness, knowledge, habits, improvisation | performance feedback, hierarchy |
| Houghton et al 2020 [24] | Asia, Africa, Central and North America, Australia | Infection prevention and control | Rapid qualitative evidence synthesis | insecurity, fatigue, communication, training, changing guidelines | support, culture, inclusiveness, workload |
| Suliman et al 2018 [25] | Jordan | Infection prevention and control | Cross sectional survey and observation checklist | knowledge, education |  |
| Chapman et al 2017 [26] | Domenican Republic | Infection prevention and control | Semi structured interviews | beliefs, patient communication quality |  |
| Darawad et al 2012 [27] | Jordan | Hand hygiene | Cross sectional multicenter survey | beliefs, attitudes, experience, performance |  |
| Jenner et al 2006 [28] | UK | Hand hygiene | Observations & survey | beliefs |  |
| McLaws et al 2015 [29] | Iran | Hand hygiene |  | attitudes, beliefs, respect, laziness, risk, obligation, habit, personal decision, conscientiousness, training | workload, role model |
| O'Boyle, Henly, Larson 2017 [30] | USA | Hand hygiene | longitudinal observational & self-report | motivation, habits |  |
| Shah et al 2015 [31] | UK | Infection prevention and control | Interviews | experience, stress, entitlement, perceptions | power/hierarchy, group norms, workload |
| **Author, year, reference** | **Country** | **Standardization type** | **Study design** | **Individual factors** | **Group/Social Factors** |
| Wang et al 2021 [32] | N/A | Hand hygiene | Rapid review & meta-analysis | fear, risk, self-protection |  |
| Woith, Volchenkov, Larson 2012 [33] | Russia | Infection prevention and control | Focus groups interviews | knowledge, beliefs, attitudes, punishment, moral responsibility, fear, risk | cultural norms |
| Al-Faouri et al 2021 [34] | Jordan | Hospital-generic | Cross sectional survey | knowledge, experience |  |
| Flynn & Sinclair 2015 [35] | Ireland | Hospital-generic | Descriptive, case study | experience, professional judgement, autonomy |  |
| Kakeeto et al 2017 [36] | Sweden | Hospital-generic | Survey | knowledge, patient needs | lack of time, lack of staff |
| Kotzeva et al 2014 [37] | Spain | Hospital-generic | Cross sectional online survey | decision making, beliefs, habits, local adaptation |  |
| Meyer, Hill & Baker 2020 [38] | USA | Hospital-generic | Observation, structured feedback sessions (pre-post intervention assessment) | support, safe clinical practice, professional development |  |
| Nissinboim & Naveh 2018 [39] |  | Hospital-generic | Questionnaire, hospital error reporting data | autonomy, personal judgement, flexibility |  |
| Weske et al 2019 [40] | Netherlands | Hospital-generic | Online survey | beliefs |  |
| Efstathiou et al 2011 [41] | Cyprus | Hospital-generic | Focus group interviews | vulnerability, fear, anxiety, personal costs, experience reminders, perceptions, beliefs, patient safety, attitudes, beliefs, risk, protect patient/family | power/hierarchy, norms, work culture |
| Luo et al 2010 [42] | China | Hospital-generic | Self-report survey | knowledge, training attitudes, patient pressure, patient needs,  self-efficacy |  |
| Nofal, Subin, Al-Kalaldeh 2017 [43] | Jordan | Hospital-generic | Cross sectional survey | clinical experience, knowledge, attitudes |  |
| Adeniyi et al 2017 [44] | Nigeria | Single disease-specific | Cross sectional survey | knowledge | training, local adaptation |
| **Author, year, reference** | **Country** | **Standardization type** | **Study design** | **Individual factors** | **Group/Social Factors** |
| Biswas et al 2020 [45] | Bangladesh | Single disease-specific | Rapid ethnographic -clinical observations and informal interviews | beliefs, improve professional status, meet patient expectations, professional | professional authority/power, conflicts of interest |
| Chapman et al 2011 [46] | Australia | Single disease-specific | Semi structured interviews | motivation, duty, responsibility,  avoid litigation |  |
| Gransjøen et al 2018 [47] | Norway | Single disease-specific | Semi structured interviews | fear, attitudes, knowledge, perceptions, patient demands, access, insufficient guideline |  |
| Heutinck et al 2021 [48] | Netherlands | Single disease-specific | Interviews | experience, awareness, beliefs, attitude, insufficient guidelines | communication |
| Talarico et al 2020 [49] | EU | Single disease-specific | Surveys | awareness, knowledge |  |
| Halm et al 2000 [50] | USA | Single disease-specific | Chart review & survey | attitudes, experience | peer pressure |
| Chen et al 2021 [51] | China | ^1^PPE use | Telephone interviews | training, stress/distress, coping, professional growth,  preparedness |  |
| Seitz et al 2021 [52] | USA | PPE use | Cross sectional survey | attitudes, beliefs, knowledge, training, patient needs | reprimands |
| Chughtai et al 2016 [53] | Vietnam | PPE use | RCT- 3 armed + exit interviews | beliefs, attitude, experienced adverse events, patient needs | communication |
| Hu et al 2012 [54] | China | PPE use | Survey | patient needs, vaccination, beliefs, perceptions, forgetfulness | reprimands |
| Moore et al 2005 [55] | N/A | PPE use | Critical review | experience, knowledge, training, invulnerability, stress, attitudes\beliefs, professional role, performance limits, patient needs | workload, communication, feedback, social expectations |
| **Author, year, reference** | **Country** | **Standardization type** | **Study design** | **Individual factors** | **Group/Social Factors** |
| Seale et al 2014 [56] | Australia | PPE use | Semi structured interviews | attitudes, perception, discomfort, communication, training, uncertainty, patient needs |  |
| Braaf, Manias & Riley 2013 [57] | Australia | Surgery | Telephone interviews | beliefs | information access, disruption of workflow, professional authority/power |
| Gillespie et al 2016 [58] | Australia | Surgery | Observational audit and interviews (individual and focus groups) | individual knowledge, experience, stress | discipline, work relationships, joint cultural beliefs |
| Riley, Manias & Polglase 2006 [59] | Australia | Surgery | Ethnographic design | professional judgement, knowledge, experience, daily practice, professional ethics, attitudes, risk | collegial support, power/hierarchy |
| Wangoo, Ray & Ho 2016 [60] | N/A | Surgery | Systematic review | attitudes, perceptions, patient safety, professional roles | teamwork |
| Warwick et al 2019 [61] | N/A | Surgery | integrative mixed methods review | skills, knowledge, experience, attitudes, beliefs, respect, arrogance (refuse to accept error) | power/hierarchy, communication |
| Bierbaum et al 2020 [62] | N/A | Cancer | Systematic review | attitudes\beliefs, autonomy, skills, expertise, clinical judgement, local adaptation, flexibility, information access |  |
| te Boveldt et al 2015 [63] | Netherlands | Cancer | National cross-sectional case vignette online survey | confidence |  |
| Ismaila et al 2018 [64] | Nigeria | Cancer | Cross sectional prospective survey | awareness, cognition  guidelines usability |  |
|  |  |  |  |  |  |
| **Author, year, reference** | **Country** | **Standardization type** | **Study design** | **Individual factors** | **Group/Social Factors** |
| Belizan et al 2007 [65] | Argentina, Uruguay | Mother & new-born care | Individual & focus group interviews | attitudes, collaborate decision making, perceptions, information access | communication, leadership, and peer pressure |
| Muhindo et al 2021 [66] | Uganda | Mother & new-born care | Mixed methods observational study | knowledge | collegial support |
| Yevoo et al 2020 [67] | Ghana | Mother & new-born care | ethnographic design- observations, group interviews and staff meetings | knowledge, experience, patient safety, stress, psychological, patient needs, responsibility | coordination, communication,  respect, workload |
| Othman & Darawad 2020 [68] | Jordan | Medication administration | Observations and a self-reported survey | experience, perceptions, attitudes,  “guideline usability” |  |
| Uema, Kitamura & Nakajima 2020 [69] | Japan | Medication administration | Online survey | patient needs, beliefs, risk |  |
| McEwan et al 2018 [70] | UK | Falls | Observations, review of clinical records, interviews | attitudes, training\education | communication, support, staff support |
| Stenberg & Wann-Hansson 2011 [71] | Sweden | Falls | Focus group interviews | experiences, media, guilt, motivation, daily practice, risk, knowledge,  contradicting guidelines | Reprimands, teamwork discussions |
| Johnson 2017 [72] | N/A | Other | Literature review | knowledge, beliefs, attitude, experience, fear of litigation, performance anxiety, emotions, professional role, coping, family caused disruption |  |
| Thompson & Kagan 2011 [73] | US | Other | Interviews and direct observations | attitude, beliefs, experience,  local adaptation |  |
| Toxopeus et al 2019 [74] | Netherlands | Other | Interviews | knowledge, perceptions, decision-making |  |
| Debono et al 2013 [75] | N/A | Other | Scoping review | attitudes, beliefs, perceptions, physical, cognition, knowledge, morale (high/low) patient safety, patient needs, risk, autonomy | group communication,  coordination, group norms, power\hierarchy |
| **Author, year, reference** | **Country** | **Standardization type** | **Study design** | **Individual factors** | **Group/Social Factors** |
| Yami 2015 [76] | Kingdom of Saudi Arabia | other | Elicitation study & cross-sectional survey | experience, beliefs, quality of care, support, patient & family needs, risk | collegial approval |

^1^PPE: Personal protective equipment
